# HIV/AIDS information promotion at the library: creative campaigns for young adults

**DOI:** 10.5195/jmla.2019.588

**Published:** 2019-04-01

**Authors:** Hannah F. Norton, Margaret E. Ansell, Ariel Pomputius, Mary E. Edwards, Matthew Daley, Susan Harnett

**Affiliations:** Reference and Liaison Librarian (Associate University Librarian), Health Science Center Libraries, University of Florida, Gainesville, FL, nortonh@ufl.edu; Nursing and Consumer Health Librarian (Assistant University Librarian), Health Science Center Libraries, University of Florida, Gainesville, FL, meansell@ufl.edu; Health Sciences Liaison Librarian (Assistant University Librarian), Health Science Center Libraries, University of Florida, Gainesville, FL, apomputius@ufl.edu; Reference and Liaison Librarian (Associate University Librarian), Health Science Center Libraries, University of Florida, Gainesville, FL, meedwards@ufl.edu; Information Technology Specialist, George A. Smathers Libraries, University of Florida, Gainesville, FL, daleym@ufl.edu; Information Services Librarian (Assistant University Librarian), Borland Health Science Library, Health Science Center Libraries, University of Florida, Jacksonville, FL, sharnett@ufl.edu

## Abstract

**Background:**

While rates of new HIV diagnoses have gone down nationally, Florida’s HIV-positive population is growing and remains one of the largest in the country. Given this landscape, it is clear that diverse, creative, and collaborative efforts are needed to better inform the public about HIV risks, prevention, and treatment and to encourage healthy behaviors.

**Case Presentation:**

Building on previous work, librarians at the University of Florida engaged in a yearlong project to raise awareness about HIV/AIDS risks, prevention, and treatment among university students and to improve their information-seeking behaviors related to this disease. The “Creative Campaigns” project included 3 distinct elements of activity and engagement, designed to complement one another: a graphic novel contest, a social media campaign, and training for campus health care providers. The contest yielded 4 high-quality submissions, and the month long social media campaign garnered over 50,000 views and utilized Facebook ads to extend beyond the library’s typical audience. The instruction proved useful to campus counseling and wellness staff.

**Conclusions:**

Overall, the team considered the project a success in terms of reaching new audiences in new ways, and several of its components have been integrated into subsequent projects and regular operations. Exploring new methods of outreach through social media and creative formats required careful planning and the development of new skill sets amongst project team members but proved to be a rewarding way to generate engagement in the local community.

## BACKGROUND

While the annual rate of new HIV diagnoses has gone down nationally in recent years, the rate in Florida has increased and is third highest in the United States. Likewise, Florida has a high rate of individuals living with diagnosed HIV, also third highest in the United States as of 2016 [1]. Given this landscape, it is clear that diverse, creative, and collaborative efforts are needed to better inform the public about HIV risks, prevention, and treatment and to encourage healthy behaviors. Libraries, in particular, are ideal partners for community-based health information outreach. For instance, Perry describes the role of the public library in providing targeted HIV/AIDS information in the form of print and electronic resources to children, who may experience the impact of a family member with HIV [2]. Also, a National Library of Medicine (NLM)–funded project partnered the University of Illinois at Chicago libraries with community organizations and health care clinics to provide AIDS-related information resources and access, training, and support [3].

The University of Florida (UF) Health Science Center Libraries (HSCL), in partnership with local safety net providers and community organizations, previously addressed the need for high-quality, accessible HIV/AIDS information through a project funded by NLM’s HIV/AIDS Community Information Outreach Program (ACIOP). This project promoted collaboration among community partners in the form of a “Collaborating with Strangers” networking event, which helped identify local gaps in HIV/AIDS information; four short videos promoting free, local HIV screening services and authoritative information on HIV/AIDS; easy-to-read materials on HIV/AIDS information; and train-the-trainer instruction on HIV/AIDS information resources for community organizations and health care providers in Gainesville and Jacksonville [4]. The libraries also hosted the NLM traveling exhibit, “Surviving and Thriving: AIDS, Politics and Culture,” during the grant period to raise awareness of the cultural and political impact of HIV/AIDS and developed related events such as guest speaker presentations and film screenings.

Encouraged by the success of the ACIOP project, the HSCL team designed a subsequent project to target another group at risk of HIV infection: adolescents and young adults. To enhance access to high-quality HIV/AIDS resources for this population, we utilized two new techniques: graphic novels and social media marketing. Graphic novels or comics (used synonymously in this project) are an effective tool for health information education, and graphic medicine is a growing area of work [5]. In a 1994 study, Wells found that comics are the easiest format for disseminating HIV/AIDS information, particularly to individuals with low literacy [6]. Comics are also more accessible to diverse audiences [7] and allow patients, caregivers, and health professionals to share their own experiences [8, 9]. As is demonstrated in the breadth of literature related to social media, social media marketing can be a useful tool for library outreach. A successful campaign identifies specific, strategic, and measurable goals; encourages online discussion [10]; and engages students as “content creators” [11–13]. Using this evidence to design our new project allowed us to create innovative and engaging outreach strategies that were appropriate to the target audience.

## STUDY PURPOSE

While the HSCL’s ACIOP project successfully provided information resources and training in Alachua and Duval Counties, it primarily targeted community members outside of the university. Given the statistics on young adults and HIV, anecdotal reports about UF student attitudes about HIV from staff at the UF Student Health Care Center and GatorWell Health Promotion Services (e.g., the beliefs that no one at UF has HIV and that HIV is not something UF students have to worry about), and our ready access to this population, a logical next step was to focus outreach efforts on UF students and other local young adults. To complement the work of the ACIOP project, we sought and obtained internal funding through the George A. Smathers Libraries Strategic Opportunities Program to reach out to UF students and other young adults in Alachua County.

This project, “Creative Campaigns to Promote HIV/AIDS Awareness among UF Students” (Creative Campaigns project), had two objectives: (1) to enhance the access of UF students and other young adults in Alachua County to effective materials on HIV/AIDS risks, prevention, and treatment through development of a social marketing campaign and short graphic novels and (2) to improve the information-seeking behaviors of UF students regarding HIV/AIDS risks, prevention, and treatment through trainings targeted to their health care providers and educators.

## CASE PRESENTATION

The Creative Campaigns project included three distinct elements of activity and engagement that were designed to complement one another: a graphic novel contest, a social media campaign, and training for campus health care providers.

### Graphic novel contest

Comics offer an engaging format for communities to share stories about illness and health and spread accurate health information [8]. The HIV/AIDS Graphic Novel Contest invited students, staff, and faculty at UF and members of the broader Gainesville community to submit short comics about HIV/AIDS to increase awareness of reputable information on HIV/AIDS risks, prevention, and treatment. Prizes were offered for the top submissions, and all finalist submissions were compiled into a zine for distribution to ACIOP project partners and exhibited as part of a local community event on World AIDS Day.

Guidelines and permission forms for contest submissions were created with inspiration from graphic novel contests held by other institutions and guidance from UF’s scholarly communications librarian on copyright and permissions language to protect the interests of both the contestants and the HSCL. The guidelines, permission forms, and example comic books on related topics were displayed on the contest’s online resource guide. The contest was marketed to students, faculty, and staff at UF Health (hospital system and six colleges) via emails from liaison librarians, announcements on the HSCL website, electronic signage outside the library, posts on HSCL and UF Health Facebook pages and Twitter feeds, and printed posters at the library and in adjacent buildings. Contest announcements were sent to contacts at GatorWell, HealthStreet, Sequential Artists Workshop (SAW), and UF Arts in Medicine for further distribution. Likewise, print posters were located in other campus libraries, and librarians at the Architecture and Fine Arts Library and Marston Science Library distributed the email message to their patrons.

The contest began with a free workshop offered by staff at SAW, a nationally known local comics school and Creative Campaigns project partner. SAW staff led six participants (three faculty members, one student, one staff member, and one non-UF community member) in an overview of the comic creation process and how to tell stories through comics, incorporating a variety of hands-on learning activities. The director of SAW also served as a contest judge and volunteered SAW’s exhibition space for the display.

**Figure 1:**
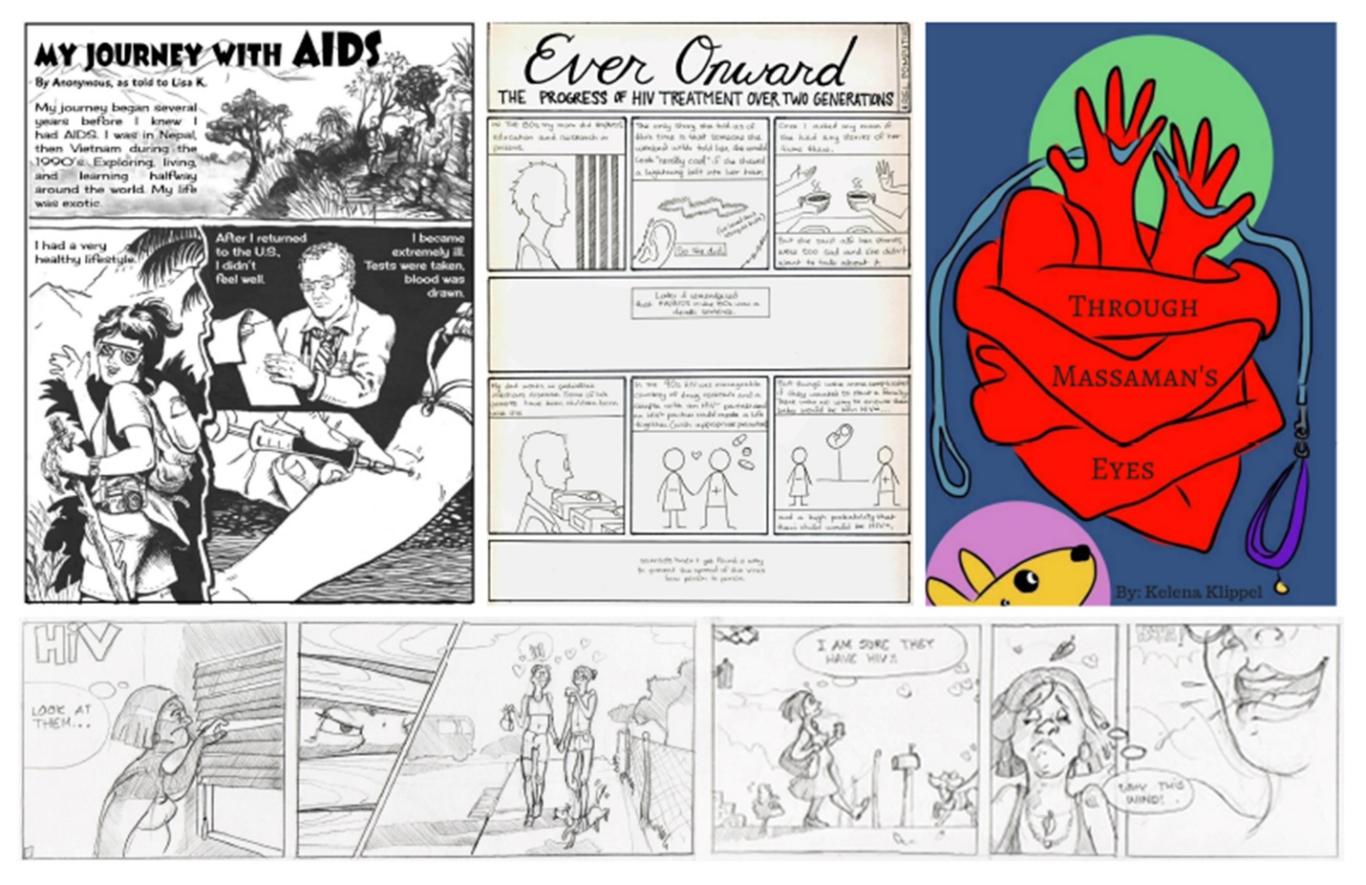
The four submissions to the HIV/AIDS Graphic Novel Contest

Four comics were submitted to the contest by 2 students, 1 faculty member, and 1 non-UF community member (Figure 1). Submissions were reviewed and evaluated by a team of 4 judges: an artist in residence at UF Health Arts in Medicine, a fine arts librarian, a staff member from GatorWell Health Promotion Services, and the director of SAW. Evaluation criteria included clarity, accuracy, and creativity of the health information message and narrative effectiveness of words and images together. All 4 submissions were deemed to be of good quality and worthy of inclusion in the zine, and 1 entry was identified as the winner. The winning creator and 2 others received monetary awards ($100 and $50, respectively).

The entries were formatted and printed in color on regular 8.5 × 11-inch paper, which was then folded in half to create a 24-page zine, *Red Ribbon Stories: Sharing Our Experiences with HIV through Comics*. An initial run of 50 zines was created for distribution, and an electronic version was uploaded to the UF Institutional Repository.

On December 1, World AIDS Day, the entries were displayed in SAW’s exhibit space as part of Artwalk Gainesville, a local community event offering a self-guided tour of art and music exhibitions in galleries, restaurants, and breweries throughout the downtown area. Full-size reproductions of the final submissions were mounted to foam boards and tacked to the display wall alongside a brief explanation of the contest and the grant-funded project. Contest participants were encouraged to attend, and all attendees were offered food and drink as well as a copy of the zine. The exhibition was advertised as part of Artwalk Gainesville, and project team members and the contest finalists were interviewed about the exhibition for the local student newspaper, the *Independent Florida Alligator*. Around eighty people visited the exhibit, and ten zines were taken. Additional copies of the zine were distributed in later weeks to campus partners (e.g., GatorWell) and local safety net health care providers.

### Social media campaign

To share HIV/AIDS narratives from the graphic novel contest broadly with students at UF and young adults in the Gainesville area, the entries were included in a month-long social marketing campaign hosted on the Facebook social media platform. The #HIVAwareUF campaign was scheduled for the month of November 2017, leading up to World AIDS Day and Artwalk Gainesville.

The campaign consisted of two types of messages: messaging promoting the project itself, including the contest entries and the Artwalk Gainesville exhibition, and messaging discussing specific issues related to HIV/AIDS, including prevention, medication, mental health, stigma and myths, HIV narratives, and research. Twenty-seven Facebook posts were created for the HSCL Facebook account, eighteen of which featured images from the graphic novel contest entries. In addition to providing a core message (e.g., “know your HIV status!”), each post included links to relevant and trustworthy resources, both local (e.g., HIV videos created during the ACIOP project) and national (e.g., factsheets from the Centers for Disease Control and Prevention and HIV.gov). Facebook pages of affiliated or relevant organizations were also tagged to direct viewers to additional resources, and unique shortened uniform resource locators (URLs) were created for the finalist entries in the graphic novel contest using the goo.gl URL shortening service to track access through the campaign.

After we collaboratively drafted the Facebook posts, five posts were selected to be tested for clarity, effectiveness, and possible offensiveness among a sample of college students using a simple three-question survey (supplemental appendix). Fifty students were recruited for testing through in-person solicitation at a campus plaza with heavy student traffic. The recruitment strategy and survey questions were developed in consultation with GatorWell staff, who frequently develop and test social media campaigns focusing on health topics. While five of the fifty students who were surveyed misunderstood the content of the posts (answering that the posts were about heart health, health in general, or “bettering the lives of others”), no students found them to be offensive. Five students gave additional positive commentary about the messages, saying that they “liked the pictures” or mentioning that they found the messages “eye-catching” or “attention-getting.” One student simply pointed to their favorite message and said, “Amen!”

To reach out to new audiences previously unaffiliated with the library, we used grant funding to pay for Facebook ads for 12 of the posts: a campaign kickoff post, a post introducing the graphic novel contest, 4 posts profiling the graphic novel contest finalists, 3 posts highlighting HIV videos that the library created for the ACIOP project, a post sharing a print flyer with a list of HIV support groups in the area (also created during the ACIOP project), and 2 posts advertising the Artwalk Gainesville exhibition. Each “boosted” post cost a maximum of $50 and was targeted to 2 specific audiences: individuals 18–30 years of age who listed their school as the University of Florida and individuals 18–30 years within 50 miles of Gainesville, Florida.

The campaign garnered 52,149 views, 5,237 of which were organic (that is, not the result of Facebook ads) (Table 1). Engagement with campaign posts was also high, with 1,120 clicks, 430 likes, 29 shares, and 19 comments. Approximately 3% of viewers engaged with campaign posts in some way (compared with higher education and nonprofit averages of <1% [14]). The average number of views of Creative Campaigns posts (n=1,324) was significantly higher than that of HSCL Facebook page posts, both 6 months before (n=176) and 6 months (n=88) after the campaign (Kruskal-Wallis test, *H*=16.2, *p*<0.01).

**Table 1:**
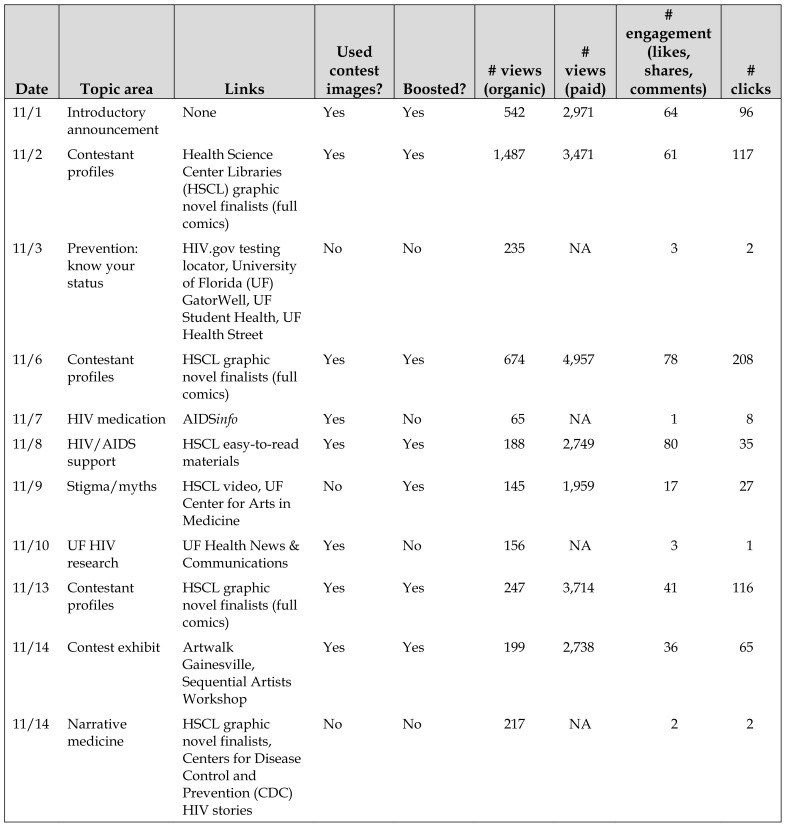

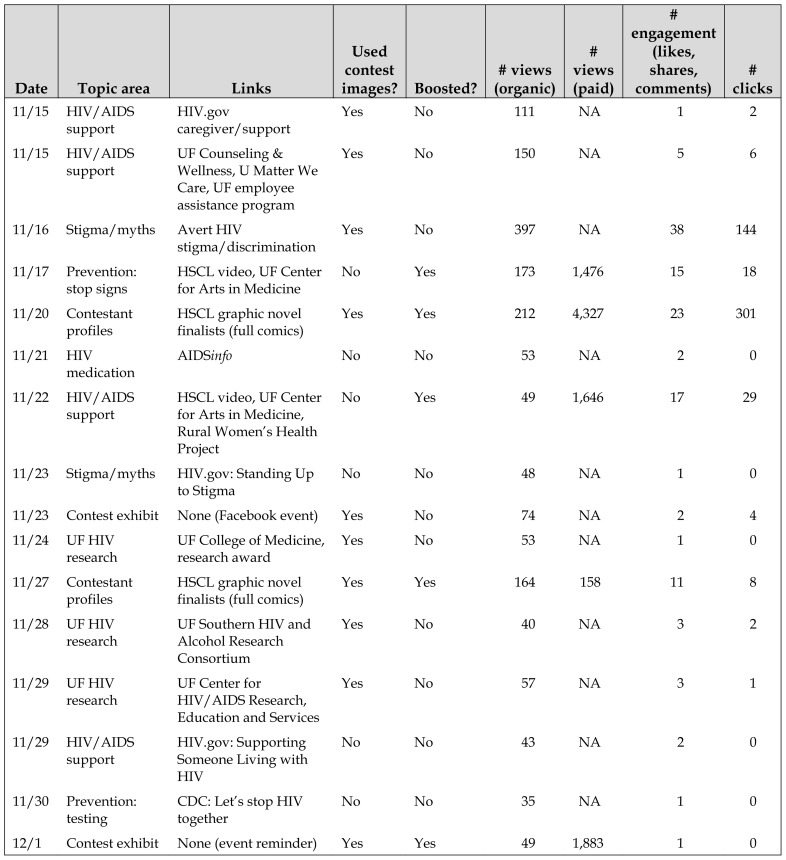
Social media campaign posts, content, views, and engagement

Date	Topic area	Links	Used contest images?	Boosted?	# views (organic)	# views (paid)	# engagement (likes, shares, comments)	# clicks
11/1	Introductory announcement	None	Yes	Yes	542	2,971	64	96
11/2	Contestant profiles	Health Science Center Libraries (HSCL) graphic novel finalists (full comics)	Yes	Yes	1,487	3,471	61	117
11/3	Prevention: know your status	HIV.gov testing locator, University of Florida (UF) GatorWell, UF Student Health, UF Health Street	No	No	235	NA	3	2
11/6	Contestant profiles	HSCL graphic novel finalists (full comics)	Yes	Yes	674	4,957	78	208
11/7	HIV medication	AIDS*info*	Yes	No	65	NA	1	8
11/8	HIV/AIDS support	HSCL easy-to-read materials	Yes	Yes	188	2,749	80	35
11/9	Stigma/myths	HSCL video, UF Center for Arts in Medicine	No	Yes	145	1,959	17	27
11/10	UF HIV research	UF Health News & Communications	Yes	No	156	NA	3	1
11/13	Contestant profiles	HSCL graphic novel finalists (full comics)	Yes	Yes	247	3,714	41	116
11/14	Contest exhibit	Artwalk Gainesville, Sequential Artists Workshop	Yes	Yes	199	2,738	36	65
11/14	Narrative medicine	HSCL graphic novel finalists, Centers for Disease Control and Prevention (CDC) HIV stories	No	No	217	NA	2	2
11/15	HIV/AIDS support	HIV.gov caregiver/support	Yes	No	111	NA	1	2
11/15	HIV/AIDS support	UF Counseling & Wellness, U Matter We Care, UF employee assistance program	Yes	No	150	NA	5	6
11/16	Stigma/myths	Avert HIV stigma/discrimination	Yes	No	397	NA	38	144
11/17	Prevention: stop signs	HSCL video, UF Center for Arts in Medicine	No	Yes	173	1,476	15	18
11/20	Contestant profiles	HSCL graphic novel finalists (full comics)	Yes	Yes	212	4,327	23	301
11/21	HIV medication	AIDS*info*	No	No	53	NA	2	0
11/22	HIV/AIDS support	HSCL video, UF Center for Arts in Medicine, Rural Women’s Health Project	No	Yes	49	1,646	17	29
11/23	Stigma/myths	HIV.gov: Standing Up to Stigma	No	No	48	NA	1	0
11/23	Contest exhibit	None (Facebook event)	Yes	No	74	NA	2	4
11/24	UF HIV research	UF College of Medicine, research award	Yes	No	53	NA	1	0
11/27	Contestant profiles	HSCL graphic novel finalists (full comics)	Yes	Yes	164	158	11	8
11/28	UF HIV research	UF Southern HIV and Alcohol Research Consortium	Yes	No	40	NA	3	2
11/29	UF HIV research	UF Center for HIV/AIDS Research, Education and Services	Yes	No	57	NA	3	1
11/29	HIV/AIDS support	HIV.gov: Supporting Someone Living with HIV	No	No	43	NA	2	0
11/30	Prevention: testing	CDC: Let’s stop HIV together	No	No	35	NA	1	0
12/1	Contest exhibit	None (event reminder)	Yes	Yes	49	1,883	1	0

Likewise, average engagement was significantly higher on campaign posts (n=38) than on HSCL Facebook page posts before (n=7) and after (n=8) the campaign (Kruskal-Wallis test, *H*=6.12, *p*=0.05). The links to the graphic novel contest finalists’ full entries were clicked 297 times through the campaign posts alone. Facebook’s algorithm for acceptable ads (favoring colorful images and video over text) caused issues with one of the boosted posts that featured the title page of one of the graphic novel finalists: because it was mostly calligraphic text, it was not shared with as many Facebook users as the other boosted posts. Some of the contest participants engaged with the HSCL Facebook account or championed the campaign to others. A few posts in the campaign caused confusion and annoyance to some Facebook users as evidenced by some comments left under the posts (e.g., “Why I keep getting aids post[sic]. I don’t have that”), but most reactions were positive.

### Training for health care providers

Another component of the Creative Campaigns project that built off work done in the HSCL ACIOP project was training for health care providers. The ACIOP project used a train-the-trainer approach to provide instruction for health care professionals (including local public health workers and safety net clinic volunteers), public librarians, and the general public related to reliable information resources on HIV/AIDS [4]. Evidence shows that train-the-trainer instruction targeted at health professionals can be effective for disseminating learned information to both other health professionals and patients [15, 16]. Because assessments during the ACIOP project illustrated that respondents were aware of only a few of the most common resources, the training highlighted a variety of additional resources. Each resource was presented in detail, with information about features, strengths, weaknesses, primary audiences, literacy level, overall layout, and navigability.

When designing the Creative Campaigns project for this new target audience, we decided to highlight the mental health aspects of living with HIV/AIDS, including addressing potential behavioral or psychosocial comorbidities and information resources related to these aspects of the disease. All of the resources highlighted from the prior project were included in the training content to provide a general foundation, but the mental health content in each was the focus of the session. The training component for this project targeted faculty and staff in the UF Counseling and Wellness Center, the designated mental health resources for UF students. Delivering the session in their facilities helped to increase attendance, and approximately thirty-five to forty faculty and staff attended. The session itself consisted of an hour-long presentation by project personnel with a combination of pre-created slides and live searching and browsing of the highlighted resources. While a training evaluation was available online to all session attendees and a follow-up reminder was sent after the session, only one participant completed the questionnaire. The one received evaluation indicated that the presented resources were new to the respondent and useful for their work.

## DISCUSSION

While the HIV/AIDS Graphic Novel Contest elicited a small number of submissions, we considered the contest a success given the quality of submissions and their applicability and versatility for use in the social media campaign. This experience emboldened the library to hold another graphic novel contest in the summer of 2018, this time focusing on the topic of addiction in connection with the NLM traveling exhibit, “Pick Your Poison: Intoxicating Pleasures and Medical Prescriptions.” We anticipated that the topic of addiction would resonate with a wider audience, thus broadening the potential participant pool. For this second iteration of the graphic novel contest, the HSCL again partnered with SAW to hold introductory workshops on comics creation; however, the workshops took place at the public library’s headquarters branch and in the HSCL, which were better known event venues for the target audience than the SAW facility that was used in the Creative Campaigns project.

Several logistical issues arose during the graphic novel contest that could be improved on in other contest implementations. Lack of restrictions on contest entry formats resulted in submissions of very different styles (black and white versus color, inked versus pencil only, landscape versus portrait orientation) that presented challenges when creating the printed zine. Handwritten entries were difficult to read once digitized, and not all entries fit easily within the zine’s 8 × 5-inch format. We recommend that others who are considering a graphic novel or other art contest define contest submission guidelines with a specific aspect ratio and emphasize that the creator must verify that any text in the submission is legible at smaller sizes, because it might be scaled down in production. This recommendation has been applied to the addiction graphic novel contest, with suggestions in the submissions guidelines to use full color or grayscale ink and information about the approximate dimensions of the resulting zine.

Likewise, the team considered the social media campaign beneficial to the library and the target audience, with more views and engagement on these posts on average than any others on the HSCL Facebook page. This high-visibility campaign helped build the library’s Facebook following during the first semester of a newly developed social media calendar: an additional 91 individuals liked the HSCL Facebook page during this month (a 16% increase over the previous month). Facebook’s ad mechanism was very effective overall, allowing audience customization and broad exposure. The campaign also served as a means for the HSCL to engage and connect with other campus entities on Facebook. Tagging GatorWell and the UF Student Health Care Center, for example, has become a standard HSCL strategy for certain types of messages beyond this campaign.

Some specific logistical challenges in the social media campaign could be improved on in future campaigns. It was sometimes difficult to transform full comics into individual social media–appropriate images with captions; many panels did not make sense without context. Having a wider diversity of contest submissions (using the strategies described above to solicit broader contest participation) may minimize the impact of having some that require more narrative context. Additionally, engagement with the posts was not always positive or appropriate, requiring active page moderation.

While HSCL faculty and staff considered the promotion of health and targeted information outreach as central to the library’s mission, it was possible that some viewers saw the HIV/AIDS campaign as tangential to the library’s core functions and, thus, outside the range of their expected interactions with the HSCL Facebook page. Finally, several campaign posts received very little engagement. This could be partially explained by Facebook’s ads mechanisms that favored image over text and were sensitive to the length of time that an ad runs. For one post, the image was calligraphy of the graphic novel entry title, which Facebook interpreted as text and, as a result, did not aggressively promote. The campaign’s last ad was set for only twenty-four hours, as it was an advertisement for the time-sensitive Artwalk Gainesville event. Because Facebook’s ad algorithm was unable to reach our target audience within that time period, that ad also underperformed. Another potential explanation for lower engagement on some posts was the length of the overall campaign: over the course of a month, the campaign might have exhausted the interest and engagement of viewers.

While instruction was a relatively small part of the Creative Campaigns project, we believe that extending instruction to focus on mental health issues surrounding HIV/AIDS was a worthwhile endeavor. The slides from this presentation were added to the HSCL HIV/AIDS guide, and the presentation served as a first connection with UF Counseling & Wellness, which might benefit from other consumer health information training in the future and has become another campus office with which to share information about relevant programming (e.g., activities related to the “Pick Your Poison” exhibit). One major limitation of the project was the lack of evaluations obtained from training participants. While the evaluation survey link was shared in the training session and a follow-up reminder sent via email, this venue might have been better suited to paper evaluations, as experienced during the HSCL’s ACIOP project [4].

A limitation of the Creative Campaigns project was its focus on health information awareness without explicit evaluation of how such awareness impacted the health behaviors of the target population: UF students and other young adults in Alachua County. While it is notoriously difficult for health information outreach projects to assess long-term impacts on health behavior, there is evidence that health knowledge, health beliefs, emotions, and social norms contribute collectively to changes in health behaviors and outcomes [17]. Additionally, Whitney and colleagues suggest that information outreach projects that address institutional change are more effective than those that simply target a specific cohort in the institution [17]. The train-the-trainer element of the Creative Campaigns project represents some initial steps in that direction.

Another limitation was the indirect means through which the project targeted improving students’ information-seeking behaviors. The train-the-trainer outreach to students’ health care providers was designed to influence providers to share with students what HIV/AIDS information resources were available and how to use them. Additionally, social media posts and the zine included a plethora of links to authoritative HIV/AIDS resources, and providing this information to students might have indirectly affected their information-seeking behaviors by targeting early steps in the research process (identifying reliable sources). However, both of these methods better served the objectives of enhancing information access and raising awareness rather than directly improving information-seeking behaviors.

Using both new techniques as well as more time-tested tools like train-the-trainer information literacy instruction, the project engaged our audience in learning more about HIV/AIDS risks, prevention, and treatment. For other libraries interested in health information outreach, social media campaigns for health promotion, or graphic medicine, we offer the following lessons learned:

- When partnering with nonlibrary groups, use their best practices to inform your work.
- A graphic novel contest can produce reputable health messages contributed by the target community.
- Common health conditions or broad health topics may solicit more user-generated content than less common or stigmatized conditions or diseases.
- For user-generated content, consider the final format for content sharing and set submission guidelines accordingly.
- Facebook ads can be an effective way for libraries to broaden their social media audience on select messages.
- Stay abreast of social media platforms’ content preferences when developing messages.
- Libraries can develop social media campaigns on specific health topics. However, a month may be too long for such a campaign focused on a single disease.
- Participants in one-time workshops may be more likely to complete print evaluations than electronic evaluations.
- For health information outreach projects, consider extending evaluation efforts beyond reach, satisfaction, and post-training response to look at longer-term changes in attitudes and behaviors.
- Finally, be creative and try new outreach methods.

Overall, we considered the project a success in terms of reaching new audiences in new ways, and several of its components have been integrated into later HSCL projects and regular operations. Exploring new methods of outreach through social media and creative formats required careful planning and the development of new skill sets amongst project team members but proved to be a rewarding way to generate engagement with the local community. Additionally, the project helped expand practical knowledge about how librarians can use creative methods to participate in awareness and information literacy campaigns that focus on health topics.

## SUPPLEMENTAL FILE

Appendix Message testing images and questions
