# Supplementary material for: HIV/AIDS information promotion at the library: creative campaigns for young adults
Source: J Med Libr Assoc. 2019 Apr 1;107(2):222–31. doi: 10.5195/jmla.2019.588 (PMC6466483; doi:10.5195/jmla.2019.588)
Supplement: Appendix [file jmla-107-222-s001.pdf]

JMLA Journal of the Medical Library Association

SC
Supplemental content to
J Med Libr Assoc. Apr;107(2):dx.doi.org/10.5195/jmla.2019.588
www.jmla.mlanet.org
© Norton, Ansell, Pomputius, Edwards, Daley, Harnett 2019

HIV/AIDS information promotion at the library: creative campaigns for young adults

Hannah F. Norton, AHIP; Margaret E. Ansell, AHIP; Ariel Pomputius; Mary E. Edwards; Matthew Daley; Susan Harnett, AHIP

APPENDIX

Message testing images and questions

Get to know the 3 types of HIV treatment (as illustrated by Kelena Klippel): ART, PrEP, and PEP! Antiretroviral therapy (ART) is a combination of drugs that keeps HIV+ people healthy, pre-exposure prophylaxis (PrEP) helps prevent people at risk of infection from getting HIV, and post-exposure prophylaxis (PEP) can help keep someone recently exposed to HIV from getting infected. Learn more about ART here: aidsinfo.nih.gov/understanding-hiv-aids/fact-sheets/21/51/hiv-treatment--the-basics. #HIVAwareUF

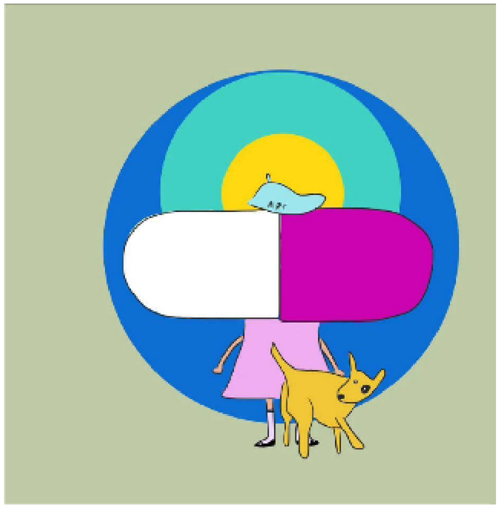

This is taken to stop the virus from growing and multiplying.

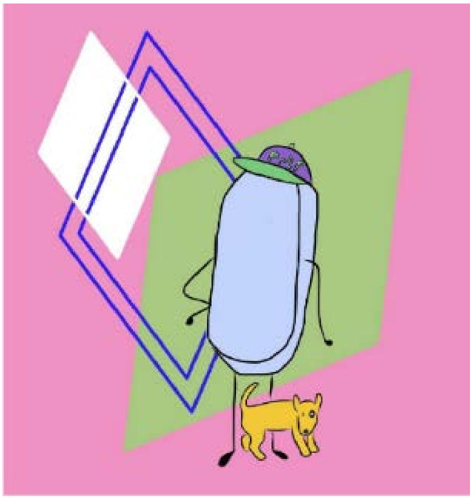

This for for individuals who are HIV negative but are at risk.

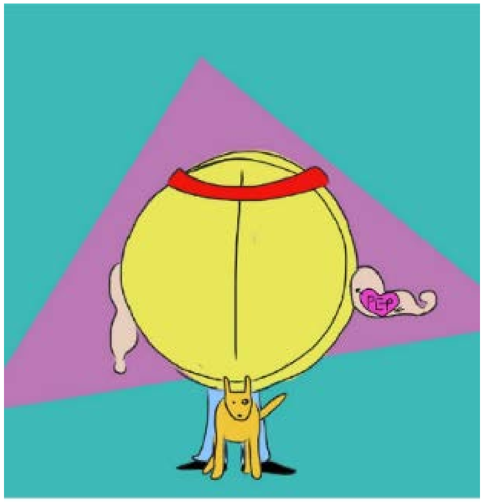

This must be taken within 72 hours of exposure.

JMLA Journal of the Medical Library Association

SC
Supplemental content to
J Med Libr Assoc. Apr;107(2):dx.doi.org/10.5195/jmla.2019.588
www.jmla.mlanet.org
© Norton, Ansell, Pomputius, Edwards, Daley, Harnett 2019

Everyone's experience with HIV/AIDS is different: we all have our own HIV story to tell. By sharing our stories, we help fight the stigma of HIV. Each of our HIV/AIDS Graphic Novel Contest entries tells a unique story. Read their stories here: guides.uflib.ufl.edu/graphic-novel-contest/finalists-HIV-AIDS, or watch the Centers for Disease Control and Prevention (CDC) video series of HIV stories here: www.cdc.gov/actagainstaids/campaigns/lsht/hiv-stigma-stories/. #HIVAwareUF

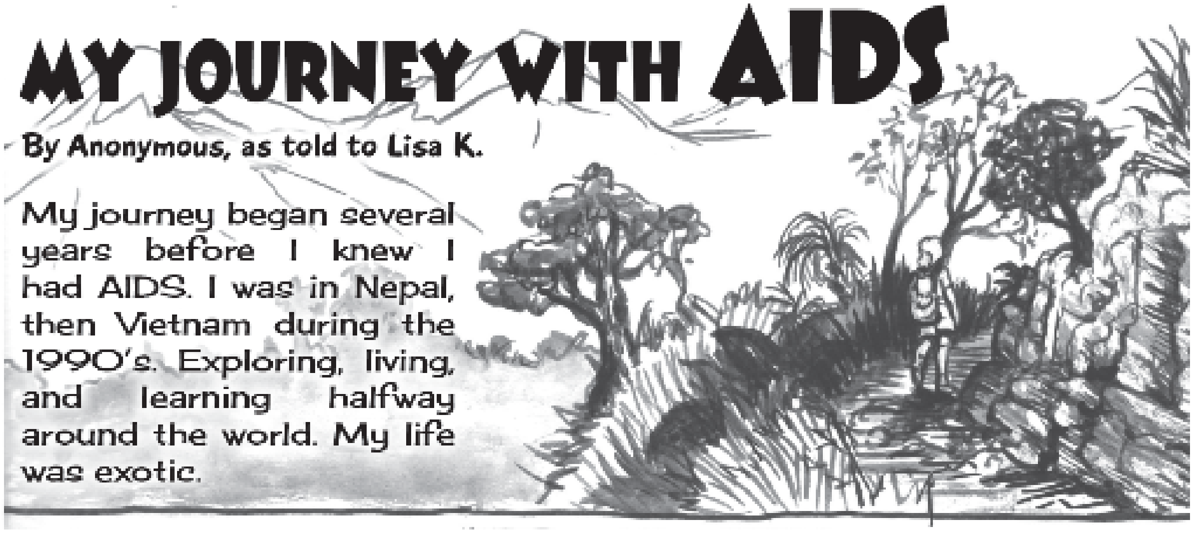

JMLA Journal of the Medical Library Association

Supplemental content to
J Med Libr Assoc. Apr;107(2):dx.doi.org/10.5195/jmla.2019.588
www.jmla.mlanet.org
© Norton, Ansell, Pomputius, Edwards, Daley, Harnett 2019

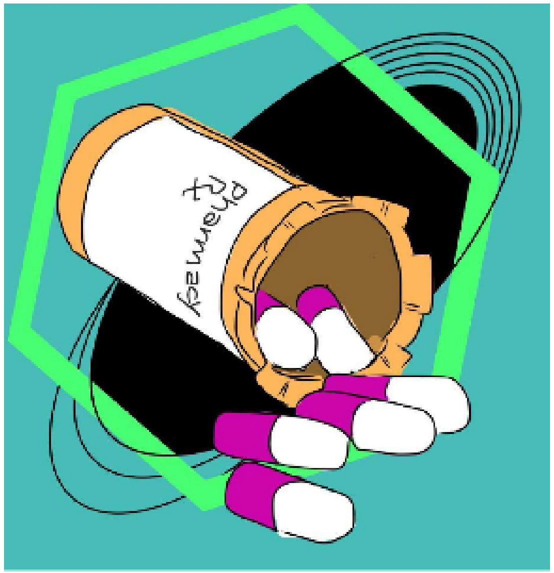

Granted, we made adjustments to uphold his quality of life.

In addition to looking for a vaccine for HIV, scientists at @ufdrgator are working to improve the quality of life of those living with HIV. This year, Carla Mavian won an award from the University of Florida (UF) to research new ways to measure the presence of HIV that do not require a blood test. Learn more about Dr. Mavian's research and the award here: pathology.ufl.edu/2017/01/23/postdoctoral-associate-carla-mavian-ph-d-wins-the-2017-thomas-h-maren-junior-investigator-postdoctoral-award/. #HIVAwareUF

If your friends or loved ones have recently been diagnosed with HIV, you can support them by being available to talk, listening and offering help, learning more about the virus, and encouraging treatment and medication adherence. You are an important and valuable part of creating a supportive and welcoming space. www.hiv.gov/hiv-basics/overview/making-a-difference/supporting-someone-living-with-hiv #HIVAwareUF

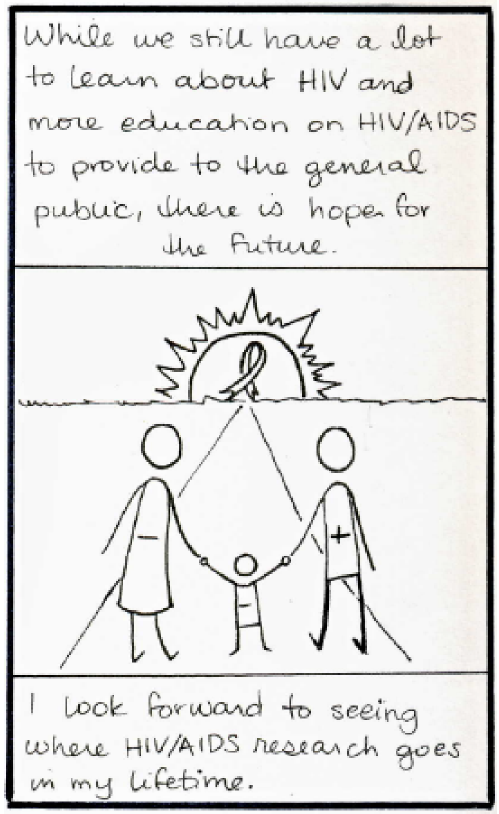

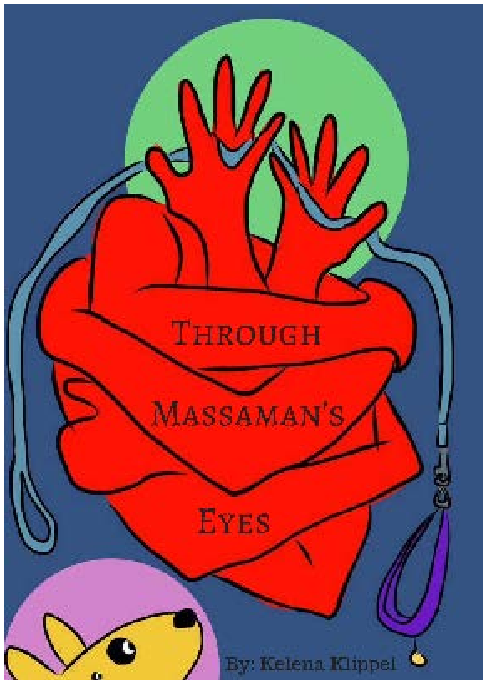

In her comic, *Through Massaman's Eyes,* Kelena Klippel demonstrates the importance of self-care and community after being diagnosed with HIV. Check out her full HIV/AIDS Graphic Novel Contest entry here: guides.uflib.ufl.edu/ld.php?content_id=36303508. #HIVAwareUF

JMLA Journal of the Medical Library Association

SC

Supplemental content to
J Med Libr Assoc. Apr;107(2):dx.doi.org/10.5195/jmla.2019.588
www.jmla.mlanet.org
© Norton, Ansell, Pomputius, Edwards, Daley, Harnett 2019

HIV/AIDS social marketing campaign

1. I think these posts are (select all that apply):

▢ Informative
▢ Offensive
▢ Entertaining
▢ Boring

2. I am motivated by these messages to learn more.

o Yes
o No

3. These posts are about:

____
